# Risk Factors for Cesarean Delivery following Labor Induction in Multiparous Women

**DOI:** 10.1155/2013/820892

**Published:** 2013-01-14

**Authors:** Corine J. Verhoeven, Cedric T. van Uytrecht, Martina M. Porath, Ben Willem J. Mol

**Affiliations:** ^1^Department of Obstetrics & Gynecology, Máxima Medical Centre, P.O. Box 7777, 5500 MB Veldhoven, The Netherlands; ^2^Department of Obstetrics & Gynecology, Academic Medical Centre, Meibergdreef 9, 1105 AZ, Amsterdam, The Netherlands

## Abstract

*Objective*. To identify potential risk factors for cesarean delivery following labor induction in multiparous women at term. *Methods*. We conducted a retrospective case-control study. Cases were parous women in whom the induction of labor had resulted in a cesarean delivery. For each case, we used the data of two successful inductions as controls. Successful induction was defined as a vaginal delivery after the induction of labor. The study was limited to term singleton pregnancies with a child in cephalic position. *Results*. Between 1995 and 2010, labor was induced in 2548 parous women, of whom 80 had a cesarean delivery (3%). These 80 cases were compared to the data of 160 parous women with a successful induction of labor. In the multivariate analysis history of preterm delivery (odds ratio (OR) 5.3 (95% CI 1.1 to 25)), maternal height (OR 0.87 (95% CI 0.80 to 0.95)) and dilatation at the start of induction (OR 0.43 (95% CI 0.19 to 0.98)) were associated with failed induction. *Conclusion*. In multiparous women, the risk of cesarean delivery following labor induction increases with previous preterm delivery, short maternal height, and limited dilatation at the start of induction.

## 1. Introduction

Induction of labor is a common and essential element of the contemporary obstetric practice and now accounts for approximately 20% of all deliveries [1–3]. Induction of labor is thought to be associated with an increase in the risk of cesarean delivery both for nulliparous and multiparous women [4]. This has been demonstrated both for inductions on medical grounds and for elective inductions [5, 6]. More recent randomized comparisons have demonstrated that the effect of the induction of labor on the risk of cesarean delivery is limited. In postterm women as well as in women with prolonged rupture of membranes at term and in women with hypertensive disease, induction of labor is more effective than expectant management [7–9].

Data in parous women undergoing labor induction have revealed conflicting results. Some observational studies suggest that the rate of cesarean delivery in multiparous women with an elective induction is similar to that in those women with a spontaneous onset of labor [6, 10, 11]. Other studies report an increased risk for those who were electively induced [12–15]. One recent study even reported a lower cesarean delivery rate in multiparous women in whom labor was induced preventively, in order to ensure that pregnant women entered labor at an optimal time for the mother-baby pair [16]. 

Not much is known about factors related to a cesarean delivery after induction of labor in multiparous women.

In women where cesarean delivery is required, the procedure not only carries the operative risks in the index pregnancy, but also increases risks for future pregnancies [17]. Consequently, it would be useful to understand which factors are related to a cesarean delivery after induction of labor in multiparous women. In view of this question, we performed a case-control study to examine which factors are associated with a failed induction of labor in multiparous women.

## 2. Methods

We performed a case-control study in the Department of Obstetrics and Gynecology of the Máxima Medical Centre in Veldhoven, in the period 1995–2010. The Máxima Medical Centre is a large teaching hospital with a regional function for tertiary perinatal care in the south of The Netherlands. We included multiparous women with a singleton pregnancy above 37 weeks in whom labor was induced. Women with a pregnancy with a known fetal anomaly or a fetus in noncephalic presentation were excluded. Cases were defined as multiparous women with at least one previous vaginal delivery, in whom labor was induced and had resulted in a cesarean delivery. For each case, we selected two multiparous women with successful inductions of labor as controls, one successful induction immediately before a case and one immediately after a case. Successful induction of labor was defined as achieving a vaginal delivery any time after the onset of induction of labor.

Induction of labor was performed according to a standard protocol. Patients with an unfavorable cervix (Bishop score < 6) received 2 mg Prostin gel (1 mg if there was prelabor rupture of membranes) in the posterior fornix of the vagina, repeating the dose after 6 hours if necessary, depending on the Bishop score (Prostin E2, dinoprostonum 1 mg/3 g (RVG 13823) or 2 mg/3 g (RVG 13824), Pfizer B.V., Capelle aan de IJssel, The Netherlands). Amniotomy was performed in women with a favorable cervix (Bishop score ≥ 6). Oxytocin augmentation was started in cases with unsatisfactory progress of labor or when contractions were absent 60 minutes after amniotomy. Continuous monitoring of fetal heart beat and uterine contractions was registered on the cardiotocograph in all cases. The analgesics used were mostly pethidine or epidural analgesia, the latter as a continuous infusion of 0.1% Ropivacaine and 0.5 *μ*g/mL Sufentanil. Cesarean delivery was performed as necessary for failure to progress in labor or for fetal distress, as assessed by cardiotocography and/or fetal blood sampling.

For cases and controls, we collected maternal age, height, body mass index (BMI), parity, reason for induction, gestational age, Bishop score, need for epidural, birth weight, and reason for cesarean delivery.

In our hospital, ethics committee approval is not required for a retrospective study.

Statistical analyses were performed using SPSS software (SPSS Inc, Chicago, IL). 

The association between potential risk indicators and the occurrence of cesarean delivery was addressed using logistic regression analysis. In the univariable analysis, we expressed the association between each variable and the occurrence of cesarean delivery with an odds ratio (OR) and a 95% confidence interval (CI). Multivariable logistic regression analysis was used to estimate the independent contribution of potential risk indicators for the occurrence of cesarean delivery. In the multivariable analysis, we entered all variables with a *P* value < .50 in the univariable analysis. Variables stayed in the model when their *P* value was < .25.

## 3. Results

**Figure 1:**
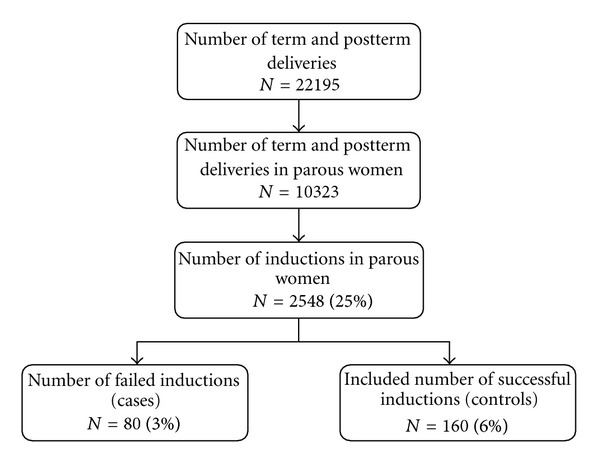
Flowchart of the study group: total number of term and postterm deliveries and inductions of labor between 1995 and 2010 in Máxima Medical Centre.

Between 1995 and 2010, labor was induced in 2548 parous women, of whom 80 had a cesarean delivery (3%). These 80 cases were compared to the data of 160 parous women who had a successful induction of labor (controls) (Figure 1). In 41 (51%) of the cases, the reason for cesarean delivery was failure to progress, in 34 (43%) fetal distress, and in 5 (6%) both.

Table 1 shows the characteristics of the study population at the start of induction of labor. There was no significant difference in maternal age, parity, or reasons for induction of labor between cases and controls. 

The characteristics of the study population during induction of labor are shown in Table 2. Prostaglandins for induction were used in 52 of the cases (65%) and in 80 (50%) of the controls (OR 1.9, 95% CI 1.1 to 3.2, *P* value 0.029). Otherwise, labor was induced by artificial rupture of membranes, mostly followed by oxytocin intravenous or by oxytocin alone in case of prolonged rupture of membranes. Furthermore, the need for epidural analgesia during labor differed between the cases and controls (OR 3.1 (95% CI 1.5 to 6.1, *P* value 0.001)). The neonates born after cesarean delivery more often had a low Apgar score after one minute (15% versus 6%) and an arterial umbilical pH under 7.15 (15% versus 8%). These children were more often admitted to the neonatal care (20% versus 7%) (OR 3.4 (95% CI 1.5 to 7.7), *P* value 0.004) (Table 3).

Cesarean delivery was significantly associated with gestational age at delivery (OR 0.97 (95% CI 0.95 to 0.99) per day), BMI (OR 1.1 (95% CI 1.01 to 1.14) per BMI point), and maternal height (OR 0.92 (95% CI 0.88 to 0.97) per cm).

Furthermore, dilatation (OR 0.64 (95% CI 0.50 to 0.83)) for every centimeter more dilated at the start of induction, nonengagement of the fetal head (OR 3.1 (95% CI 1.1 to 8.4)), and the need for using prostaglandins as induction method (OR 1.9 (95% CI 1.1 to 3.2)) were all associated with the risk of cesarean delivery. The amount of effacement of the cervix at the start of the induction did not differ between the cases and controls. In terms of previous obstetric history, women with only previous preterm delivery had a significantly higher risk of cesarean delivery than those with at least one previous term delivery (OR 3.5 (95% CI 1.8 to 6.9)). No difference in birth weight was observed between the two groups.

In the multivariable analysis (Table 4), the risk of cesarean delivery was significantly associated with low maternal height with an adjusted OR of 0.87 (95% CI 0.80 to 0.95, *P* = 0.002), a history of preterm delivery (adjusted OR 5.3 (95% CI 1.1 to 25), *P* = 0.042) and the amount of dilatation at the start of induction of labor (adjusted OR 0.43 (95% CI 0.19 to 0.98), *P* = 0.043).

In the cesarean delivery group, there were ten women (13%) with the presenting fetal head at the level of the pelvic inlet, so at station -3. Indications for cesarean delivery were failure to progress because of neglected transverse lie (*n* = 1), prolapsed fetal arm (*n* = 3), prolapsed umbilical cord (*n* = 1), non-engaged persistent occiput anterior position (*n* = 1), failure to progress of dilatation (*n* = 2) and fetal distress (*n* = 2). In 50% of them, the method of induction was amniotomy, mostly followed by oxytocin. In the control group, in seven women (4%) the presenting fetal head at the start of the induction of labor was at station -3. In only 29% (2/7) of these seven women amniotomy was performed, and they all delivered spontaneously. 

We also analyzed our data stratified for the reason for cesarean delivery: failure to progress or fetal distress. There were no significant differences between those groups. Both in the univariable and the multivariable regression analysis the point estimates of the odds ratios were approximately the same as in the analysis for the whole group.

## 4. Discussion

In our hospital, we found that induction of labor in multiparous women resulted in a cesarean delivery rate of 3%. This study demonstrates that maternal height, the amount of dilatation at the start of induction and a history of preterm delivery played significant roles in determining the risk of cesarean delivery in induced multiparous women.

In this study, in which we only included women with at least one previous vaginal birth, we considered the need for a cesarean after induction of labor in a multiparous woman as the outcome measure failure of induction of labor. Although a woman might have reached active labor, we were not interested in another definition of failed induction, such as not reaching vaginal delivery within 24 hours. Although the latter outcome is frequently used, we consider it as less relevant, since in our opinion the birth of a healthy child from a healthy mother as well as spontaneous delivery, are of more importance than a quick delivery.

Women with a previous preterm delivery had a higher risk of cesarean delivery after induced labor than those with at least one previous term delivery. This finding corresponds with the results of the study of Park et al. [18]. He examined the predictive value of previous obstetric history, Bishop score, and sonographic measurement of cervical length for predicting failed induction of labor in parous women at term. Induction failed in 15 women (14%) of whom 13 delivered vaginally after 24 hours and two had a cesarean delivery (1.8%). The group of women with only previous mid trimester loss or preterm delivery had a significantly higher risk of failed labor induction than the group with at least one previous term delivery (50% (3/6) versus 12% (12/103); *P* = 0.033). Our results are in line with the results of Park, indicating that the course of induction in women with a history of preterm delivery differs from women with a term delivery. 

In 13% (10/80) of the cases, the fetal head at the start of induction was at station -3, whereas in the control group this was only 4% (7/160). Taking the reasons for cesarean delivery in these cases into account, one may conclude that one should be careful performing amniotomy if the fetal head is not properly engaged. 

Although we are aware of the additional risks inducing women with a history of cesarean delivery, we included in both cases and controls six women with a previous cesarean delivery, besides a previous vaginal delivery. A history of previous cesarean delivery was, in our study, not significantly associated with the occurrence of cesarean delivery after induction of labor.

The 3% rate of cesarean delivery in multiparous women in whom labor was induced should be compared with the cesarean delivery rate in multiparous women with spontaneous labor. Heffner reports a cesarean delivery rate of 2.4% among multiparous women with spontaneous labor [19]. The study of Jacquemyn et al. reported a cesarean delivery rate of 1.5% in women with spontaneous onset of labor, as compared to 2.8% in women with induced labor [12]. In the study of Nicholson the cesarean delivery rate was 9.9% [16].

As with other retrospective analyses, this study does have limitations of relying on data evaluated and collected in a nonstandard fashion and the inability to obtain clarification when information is not clearly delineated in the patient records. As a result of this, we did not have any information on the position of the fetal head at the start of the induction, nor had we enough information on the position and the consistency of the cervix and the weakness to enable us to calculate the Bishop score for every patient in our study. Furthermore, it would have been interesting to look at the time between deliveries to investigate whether this variable had predictive value. Unfortunately, these data were not present.

Although matching facilitates study precision, we did not match cases and controls to prevent statistical bias because of overmatching. Instead, we used multivariable regression to control for confounding.

The absolute risk of cesarean delivery after induction of labor in multiparous women is low. However, the multivariable analysis showed that this risk is increased in case of a history of preterm delivery, lower maternal height, and little dilatation at the start of induction of labor. This information will allow more accurate counseling and better informed consent in the decision-making process regarding induction of labor in multiparous women.

## Figures and Tables

**Table 1:** Characteristics of the study population at start of induction of labor.

Characteristics	Failed induction in multiparous women (*n* = 80)	Successful induction in multiparous women (*n* = 160)	Odds ratio (95% CI)	*P* value
Maternal age in years, mean (±SD)	32.5 (3.8)	32.1 (3.7)	1.1 (0.96–1.1)	0.447
Gestational age in weeks, median (range)	39^+2^ (37^+0^–42^+1^)	40^+2^ (37^+0^–42^+2^)	0.97 (0.95–0.99)	0.021
BMI in kg/m^2^, mean (±SD)	27.8 (5.3)	25.8 (5.2)	1.1 (1.01–1.14)	0.024
Height in cm, mean (±SD)	165.7 (7.3)	169.4 (6.7)	0.92 (0.88–0.97)	<0.001
Weight gain in pregnancy in kg, mean (±SD)	12.1 (5.2)	13.7 (5.4)	0.94 (0.88–1.0)	0.105
Parity				
1	52 (65%)	99 (62%)	0.87 (0.50–1.5)	0.637
≥2	28 (35%)	61 (38%)		
History of preterm delivery	21 (27%)	14 (9%)	3.5 (1.8–6.9)	0.0001
Previous cesarean delivery	6 (8%)	6 (4%)	2.1 (0.65–6.7)	0.218
Reason for induction				
Prelabor rupture of membranes	3 (4%)	8 (5%)	1.0	0.411
Hypertensive disorder	14 (18%)	24 (15%)	1.6 (0.35–6.8)	0.559
Postdates (≥42^+0^)	16 (20%)	36 (23%)	1.2 (0.28–5.1)	0.819
Maternal disease	7 (9%)	15 (9%)	1.2 (0.25–6.2)	0.789
Intrauterine growth restriction	5 (6%)	11 (7%)	1.2 (0.22–6.6)	0.824
Nonreassuring fetal heart rate	2 (3%)	1 (1%)	5.3 (0.34–83)	0.232
Other reasons	17 (21%)	18 (11%)	2.5 (0.57–11)	0.222
Maternal request	16 (20%)	47 (29%)	0.91 (0.21–3.8)	0.895

CI: confidence interval.

**Table 2:** Characteristics of the study population during induction of labor.

Characteristics	Failed induction in multiparous women (*n* = 80) (%)	Successful induction in multiparous women (*n* = 160) (%)	Odds ratio (95% CI)	*P* value
Dilatation at start induction (per cm dilation)			0.64 (0.50–0.83)	0.001
0	28 (35)	27 (17)		
1	26 (33)	46 (29)		
2	12 (15)	40 (25)		
3	7 (9)	28 (18)		
≥4	3 (4)	10 (6)		
Missing	4 (5)	7 (4)		
Effacement at start induction			0.84 (0.68–1.0)	0.098
0%	31 (39)	44 (27)		
25%	4 (5)	4 (3)		
50%	25 (31)	58 (36)		
75%	7 (9)	32 (20)		
100%	9 (11)	13 (8)		
Missing	5 (5)	9 (6)		
Stations of presentation			3.1 (1.1–8.4)	0.029
Not engaged (-3/3 station)	10 (13)	7 (4)		
Engaged	67 (84)	144 (90)		
Missing	3 (3)	9 (6)		
Method of induction				
Prostaglandins	52 (65)	80 (50)	1.9 (1.1–3.2)	0.029
AROM* and/or oxytocin	28 (35)	80 (50)		

CI: confidence interval.

*Artificial rupture of membranes.

**Table 3:** Neonatal outcome.

Characteristics	Failed induction in multiparous women (*n* = 80)	Successful induction in multiparous women (*n* = 160)	Odds ratio (95% CI)	*P* value
Birth weight in g, mean (±SD)	3493 (747.9)	3522 (605.6)	1.0 (1.0–1.0)	0.743
Apgar score after 1 minute				
<7	12 (15%)	10 (6%)	2.6 (1.1–6.4)	0.031
≥7	68 (85%)	150 (94%)		
Apgar score after 5 minutes				
<7	1 (1%)	2 (1%)	1.0 (0.09–11)	1.000
≥7	79 (99%)	158 (99%)		
pH arteria umbilicalis				
<7.15	12 (15%)	13 (8%)	1.3 (0.57–3.2)	0.512
≥7.15	59 (74%)	85 (53%)		
Missing	9 (11%)	62 (39%)		
Admission neonatal care				
Yes	16 (20%)	11 (7%)	3.4 (1.5–7.7)	0.004
No	64 (80%)	149 (93%)		

CI: confidence interval.

**Table 4:** Multivariable analysis with adjusted odds ratios indicating the risk of cesarean delivery.

Variable	Adjusted odds ratio	95% confidence interval	*P* value
Height in cm	0.87	0.80–0.95	0.002
History of preterm delivery	5.3	1.1–25	0.042
Dilatation at start induction (per cm dilation)	0.43	0.19–0.98	0.043
